# DiOHF Protects Against Doxorubicin-Induced Cardiotoxicity Through ERK1 Signaling Pathway

**DOI:** 10.3389/fphar.2019.01081

**Published:** 2019-09-27

**Authors:** Danqi Chang, Hang Li, Cheng Qian, Yanggan Wang

**Affiliations:** ^1^Department of Cardiology, Zhongnan Hospital of Wuhan University, Wuhan University, Wuhan, China; ^2^Medical Research Institute of Wuhan University, Wuhan University, Wuhan, China

**Keywords:** DiOHF, doxorubicin, cardiotoxicity, siRNA, ERK1/2

## Abstract

Doxorubicin (DOX) is an effective anticancer agent. Its clinical use is, however, limited due to its detrimental side effects, especially the cardiotoxicity caused by ROS, mitochondrial dysfunction and apoptosis. 3’,4’-dihydroxyflavonol (DiOHF) is a recently developed potent synthetic flavonoid which has been reported to exert anti-oxidative activity in myocardial ischemia–reperfusion injury and maintain the normal mitochondrial function. The aim of this study was to explore the protective effects of DiOHF on the DOX-induced cardiotoxicity. We established DOX-induced cardiotoxicity in H9C2 cells by incubation with 1 μM DOX and in BALB/c mice by DOX injection. DiOHF effectively prevented and reversed the DOX-induced cardiotoxicity, including ROS production, mitochondrial dysfunction, and apoptosis. The DOX-induced cardiotoxicity was accompanied by ERK1/2 activation and abolished by the silence of ERK1, rather than ERK2. Furthermore, DOX treatment in mice induced an increase in serum CK-MB level and myocardial fibrosis with a reduction in left ventricular (LV) function. These detrimental effects were blunted by DiOHF administration. Conclusion: DiOHF suppresses and reverses the DOX-induced cardiotoxicity by inhibiting ROS release, stabilizing mitochondrial function and reducing apoptosis through activation of the ERK1 signaling.

## Introduction

Doxorubicin (DOX), an anthracycline antibiotic, has been widely used as a chemotherapeutic agent for the treatment of various cancers in human (Todaro et al., 2013). Under some circumstances, however, its clinical use in cancer treatment has been restricted due to the drug-induced development of cardiomyopathy and congestive heart failure (Von Hoff et al., 1979; Minotti et al., 2004; Takemura and Fujiwara, 2007; Renu et al., 2018).

Previous studies have found that DOX-induced cardiotoxicity involves the production of reactive oxygen species (ROS) (Xu et al., 2001; Menna et al., 2012; He et al., 2018), mitochondrial dysfunction (Dolinsky, 2017; Xia et al., 2017; Gorini and De Angelis, 2018; Govender et al., 2018; He et al., 2018; Liu et al., 2018), and apoptosis (Wang et al., 2014; Mantawy et al., 2017; Chen et al., 2018; Tang et al., 2018), although the underlying mechanisms are unknown. Most evidence indicate that the major mechanism of DOX-induced cardiotoxicity involves ROS production (Xu et al., 2001; Menna et al., 2012; He et al., 2018). The quinone structure of DOX can participate in redox reactions as an electron acceptor, being turned into a semiquinone free radical. The semiquinone is unstable, and can provoke injury to the DNA or can be turned into quinone, which causes the production of more ROS (La Ferla et al., 2011; Thorn et al., 2011). Excessive ROS can cause oxidative stress, which refers to the imbalance between the production of ROS and the inner antioxidant system. In addition, some studies have found the changes in submicroscopic structure of organelles after DOX treatment, including the mitochondria, cytoplasm and muscle fibers, etc. (Singal et al., 2000). Furthermore, it has been reported that oxidative stress could induce the release of cytochrome c, which would activate caspase 3 in mitochondria and lead to apoptosis (Crompton, 2000). Recently, flavonoids have attracted many attentions as antioxidants (Dong et al., 2014; Mantawy et al., 2014; Sahu et al., 2016; Mantawy et al., 2017). In addition to the flavonoids extracted from plants, a few semi-synthetic and synthetic flavonoids have been reported to exert more potent antioxidative activity (Cotelle, 2001; van Acker et al., 2001; Bast et al., 2007; Luo et al., 2016; Aladedunye et al., 2017; Dos Santos et al., 2018). 3’,4’-Dihydroxyflavonol (DiOHF) is one of the synthetic flavonoids which has shown cardioprotective property in the ischemia–reperfusion injury (Wang et al., 2004; Qin et al., 2011; Woodman et al., 2014). The protective role of DiOHF is mainly due to its anti-oxidation and improvement of mitochondrial function (Wang et al., 2004; Qin et al., 2011; Woodman et al., 2014). However, whether DiOHF protects against the DOX-induced cardiotoxicity remains unknown.

It is known that the extracellular signal-regulated kinase (ERK) cascade can transmit signals from extracellular stimuli to regulate fundamental cellular functions. Thomas et al. (2015) have reported the involvement of MEK/ERK1/2 signaling in the cardioprotection of DiOHF. ERK1/2 could interact with the mitochondrial membrane and affect mitochondrial energy metabolism, autophagy, and apoptosis (Ballard-Croft et al., 2005). The underlying mechanism, however, remains unclear. In the present study, the effects of DiOHF on protection of the DOX-induced cardiotoxicity and the possible molecular mechanism were studied.

## Materials and Methods

### Drugs and Reagents

DiOHF was purchased from Indofine Chemical Company (Hillsborough, USA). Doxorubicin was obtained from Sigma (St Louis, MO, USA). Reactive Oxygen Species assay kit and mitochondrial membrane potential assay kit were obtained from Beyotime Biotechnology (Shanghai, China). Primary antibodies against cleaved-caspase-3, Bid, and Bcl-2 were obtained from Abclonal (Wuhan, China), and ERK1/2, p-ERK1/2, and GAPDH were obtained from Cell Signaling Technology (Beverly, MA, USA). The Pierce enhanced chemiluminescence (ECL) western blot substrate and BCA™ protein assay kit were purchased from Thermo Scientific (Rockford, USA). TRIzol reagent was procured from Invitrogen (Carlsbad, CA, USA). The PrimeScript reverse transcription (RT)-polymerase chain reaction (PCR) kit and SYBR Premix Ex Taq II were obtained from TransGen Biotech (Beijing, China). The primers were procured from Tianyihuiyuan (Guangzhou, China), and the siRNA constructs were obtained from RiboBio (Guangzhou, China).

### Cell Culture, Transient Transfection, and *in Vitro* Experimental Design

The rat cardiomyoblast cell line H9C2 was purchased from the American Type Culture Collection (ATCC, Manassas, VA, USA) and cultured in Dulbecco’s Modified Eagle’s Medium (DMEM, Hyclone, South Logan, UT, USA), supplemented with 10% fetal bovine serum (FBS, Gibco, Grand Island, NY, USA), L-glutamine (2 mM), penicillin (100 U/ml), and streptomycin (100 U/ml). The cells were incubated in humidified air (5% CO_2_) at 37°C. When the cells were grown to 70% confluence, ERK2/ERK1-siRNA were transfected using Lipofectamine 2000 (Invitrogen, Carlsbad, CA, USA), and cells were harvested after 48 h transfection for further experiments.

DiOHF was dissolved in DMSO and DOX was dissolved in saline. Both of them were diluted in the culture medium before the experiment. A culture medium with the same volume of DMSO was used as a control. Initially, the cells were divided into 5 groups, (i) control group; (ii) DOX (1 µM) treatment group, (iii) DiOHF (10 µM) treatment group, (iv) DiOHF + DOX group (the cells were pretreated with DiOHF for 2 h before co-incubating with DiOHF and DOX for 24 h), and (v) DOX + DiOHF group (the cells were treated with DOX for 2 h before co-incubating with DiOHF and DOX for 22 h). To further explore the protective mechanisms of DiOHF, the cells were assigned to the following groups: (i) control group (transfected with Scr-siRNA/ERK1-siRNA/ERK2-siRNA), (ii) DiOHF group (transfected with Scr-siRNA/ERK1-siRNA/ERK2-siRNA), (iii) DOX group (transfected with Scr-siRNA/ERK1-siRNA/ERK2-siRNA), and (iv) DiOHF + DOX group (transfected with Scr-siRNA/ERK1-siRNA/ERK2-siRNA). After treatment, the cells were harvested for the subsequent experiments.

### Assessment of Cell Viability

The cell viability was monitored by CCK-8 kit (Dojindo, Kumamoto, Japan) following the manufacturer’s instructions. H9C2 cells were seeded in 96-well plates, and six parallel replicates were prepared. After treating the cells as mentioned above, CCK-8 (10 µl) was added to each well and then incubated at 37°C for 2 h. The optical density (OD) was obtained by a microplate reader (Biotek Winooski, Vermont, USA at 450 nm).

### Estimation of ROS

ROS was estimated by a Reactive Oxygen Species assay kit (Beyotime Biotechnology, Shanghai, China) according to the manufacturer’s instructions. The H9C2 cells were seeded in 96-well plates. Following different treatments, the media were removed, and the cells were loaded with DCFH-DA (10 µM) at 37°C for 20 min. Subsequently, the cells were washed with clear media for three times, and the fluorescence intensity (excitation 488 nm, emission 525 nm) was detected by a fluorescent microplate reader (Molecular Devicesmd, USA).

### Apoptosis Assay

Apoptosis was analyzed by staining the cells with Annexin V and propidium iodide (Bestbio, Shanghai, China) according to the manufacturer’s instructions. The stained cells were detected within 1 h by LSRFortessa X-20 (BD Biosciences, San Jose, CA, USA), and the apoptosis rate was analyzed by Flowjo software. The cells were collected and washed twice with cold phosphate buffered saline, and then resuspended with 400 µl binding buffer. FITC Annexin V (5 µl) and propidium iodide (5 µl) were added to each tube and the tubes were left in dark for 15 min before taking the reading in a flow cytometer.

### Real-Time PCR

Total RNA was extracted from the cells using TRIzol reagent (Invitrogen, Carlsbad, CA, USA). A total of 0.5 μg of RNA samples were reverse-transcribed using the PrimeScript RT-PCR kit (Transgen Biotech, Beijing, China). Gene specific primers were shown in **Table 1**. These primers were purchased from Tianyihuiyuan (Guangzhou, China). Semi-quantitative real-time PCR was performed using SYBR Premix Ex Taq II (Transgen Biotech, Beijing, China). All data were subsequently normalized to the GAPDH mRNA level and expressed as relative mRNA expression.

**Table 1:** Primers used for real-time RT-PCRs.

Target genes	Forward primers (5’-3’)	Reverse primers (5’-3’)
ERK1	ACACTGGCTTTCTGACCGAG	GCCCACAGACCAGATGTCAA
ERK2	AGAGTGCCTTCTGACTTTCCTG	TGGAAGACCTGATGGAGACGA
GAPDH	AGTGCCAGCCTCGTCTCATA	GATGGTGATGGGTTTCCCGT

### Western Blot

The cells of each group were collected and protein was extracted. Protein extracts were prepared in the lysis buffer. The lysis buffer was a combination of RIPA, complete, phosstop, and 10 mM PMSF. A BCA™ protein assay kit (Thermo Fisher Scientific, Rockford, USA) was used to estimate the protein concentration. The protein samples were separated by SDS-PAGE and transferred onto polyvinylidene difluoride membrane (EMD Millipore). The membranes were then blocked with 5% nonfat milk or bovine serum albumin (for detecting phosphorylated protein) in TBST (Tris-buffered saline with 0.1% Tween-20) for 2 h at room temperature. Later, the membranes were incubated overnight with cleaved-caspase-3, Bid, Bcl-2, ERK1/2, p-ERK1/2, and GAPDH (diluted in dilution for primary antibodies, Servicebio, Wuhan, China) at 4°C. After washing with TBST for three times, the membranes were exposed to the corresponding secondary antibodies (1:5,000) at room temperature for 1 h. The target proteins were visualized with an ECL kit.

### Detection of Mitochondrial Membrane Potential (MMP)

MMP in H9C2 cells was determined by Mitochondrial Membrane Potential assay kit with JC-1 (Beyotime Biotechnology, Shanghai, China). Briefly, the cells were harvested and stained with JC-1 (1×) for 20 min at 37°C and analyzed with LSRFortessaX-20. The cells in the glass bottom culture dish were stained with JC-1, analyzed by Zeiss LSM 880 confocal microscope system (Carl Zeiss, Jena, Germany), and processed with ZEN software (Carl Zeiss, Jena, Germany).

### *In Vivo* Experimental Design

Female BALB/c mice weighing 20–22 g, 8–10 weeks, were purchased from Hubei Provincial Center for Disease Control and Prevention. The mice were housed in a specific pathogen free (SPF) environment at 20 ± 5°C under a 12 h light/dark cycle and provided with food and tap water. All experiments conformed to the Guide for the Care and Use of Laboratory Animals published by the US National Institutes of Health. The protocol was approved by the Animal Ethics Committee of Wuhan University.

The mice were randomly divided into 4 groups (n = 30 in each group): (i) control group (DMSO in saline, i.p.); (ii) DOX treatment group (20 mg/kg, cumulative dose of DOX, i.p.), (iii) DiOHF treatment group (6 mg/kg DiOHF daily for 10 days, i.p.), and (iv) DiOHF + DOX group (i.p. DiOHF daily for 10 days from 3 days before DOX treatment). The mortality rate in the mice was recorded for survival analysis. The remaining mice were weighed and killed after 7 days of DOX administration. Blood samples from the angular vein were collected from each mouse, and serum was separated by centrifugation at 3,800 rpm for 10 min for determination of creatine kinase MB isoenzyme (CK-MB), ALT and urea using commercial ELISA kits. For assessment of the histopathological changes, the hearts of mice from different groups were obtained, weighed, and processed for histopathological staining.

### Echocardiography

After 7 days of DOX treatment, the mice were subjected to transthoracic echocardiography. M-mode and two-dimensional echocardiography were performed with a Vevo 2100 imaging system equipped with a 30 MHz MS400 linear array transducer (VisualSonics, Toronto, ON, Canada). Left ventricular end-systolic volume (LVvols), and left ventricular internal diameter (LVID) during systole (LVIDs) and diastole (LVIDd) were estimated. The left ventricular ejection fraction (LVEF) was calculated as % EF = [(LVIDd − LVIDs)/LVIDd] × 100, and fractional shortening (FS) was calculated as % FS = [(LVIDd − LVIDs)/LVIDd] × 100.

### Histopathological Examination

The mice were sacrificed by cervical dislocation. The hearts were harvested for further morphological analysis. After fixing in 10% formalin, the hearts were embedded in paraffin. To assess the collagen deposition, tissue sections were cut transversely to obtain 4–5 mm thick sections and stained with Masson-trichrome stain. The images were obtained under a bright field microscope (Leica Aperio VERSA 8, Wetzlar, Germany) and analyzed with Image-Pro Plus 6.0.

### Statistical Analysis

The data were represented as the mean ± SEM. The mean values of more than 3 groups were compared by one-way ANOVA followed by Tukey’s multiple comparison tests. The statistical significance was set at P < 0.05.

## Results

### DiOHF Suppressed the DOX-Induced Cytotoxicity

**Figure 1:**
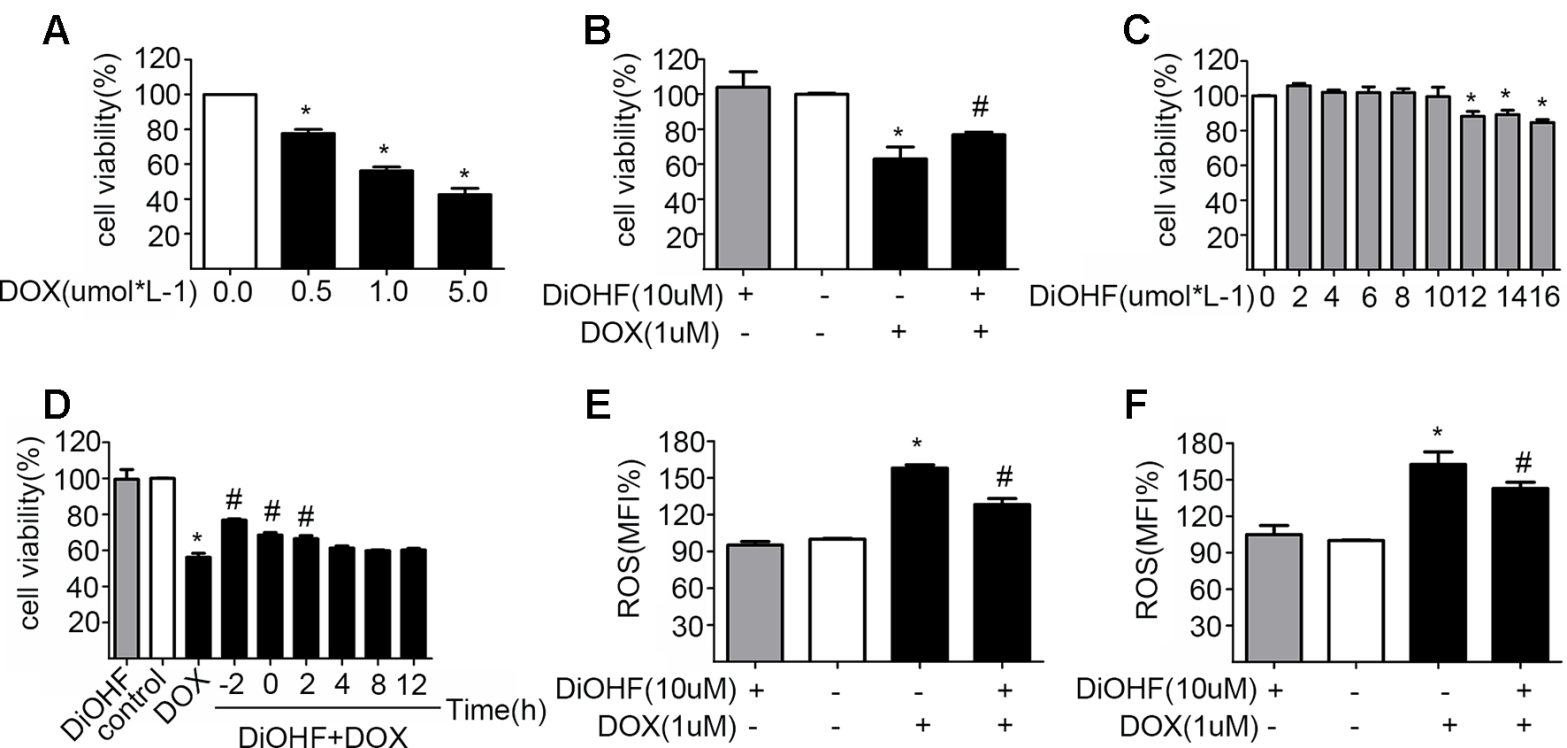
The protective effects of DiOHF on H9C2 cells against the DOX-induced cytotoxicity **(A)** DOX led to the reduction of cell viability in a dose-dependent manner (n = 3). **(B)** DiOHF inhibited the DOX-induced reduction of cell viability (n = 3). **(C)** Effects of different concentrations of DiOHF on cell viability (n = 3). **(D)** The H9C2 cells were treated with vehicle or DOX (1 µM for 24 h), and DiOHF was administrated at different time points (–2, 0, 2, 4, 8, and 12 h). Cell viability was determined using CCK-8 kit (n = 3). **(E)** Pre-treatment with DiOHF for 2 h reduced the DOX-induced ROS production (n = 3). **(F)** Post-treatment with DiOHF for 2 h also decreased the DOX-induced ROS production (n = 3). Data were analyzed by ANOVA and expressed as the mean ± SEM. *P < 0.05, compared with control. ^#^ *P* < 0.05, compared with DOX.

H9C2 cells were incubated with various concentrations of DOX for 24 h and cell viability was evaluated using CCK-8 kit to determine a suitable concentration of DOX. Cell viability was reduced significantly after DOX treatment in a dose-dependent manner (**Figure 1A**), and the DOX concentration at which the cell viability was reduced to approximately 60%, i.e. DOX concentration of 1 µM, was selected for the subsequent experiments. This particular concentration is also clinically relevant because it corroborates with the plasma concentration of DOX in the patient undergoing chemotherapy (Frost et al., 2002). The DOX-induced reduction of cell viability was largely prevented by pre-treatment with 10 µM DiOHF for 2 h (**Figure 1B**). At this concentration (≤10 µM), DiOHF alone had no effect on the cell viability (**Figure 1C**). Thus, for the subsequent *in vitro* studies, the cells were treated with 1 µM DOX for 24 h with or without 10 µM DiOHF.

### DiOHF Reversed the DOX-Induced Cytotoxicity

We have shown that DiOHF pre-treatment could prevent DOX-induced cytotoxicity. Here, we tested whether DOX-induced reduction of cell viability is reversible by DiOHF. To assess this, we first determined the time course of DiOHF effects on cell viability. After DOX treatment, cell viability (**Figure 1D**) showed a modest recovery with 2 h of post-treatment with DiOHF, whereas DiOHF could no longer rescue cell viability when the treatment with DOX exceeded 4 h. Earlier administration of DiOHF resulted in better restoration of cell viability. Thus, a 2 h post-treatment was regarded as the proper time point.

### DiOHF Inhibited DOX-Induced ROS Production

The mean of fluorescence intensity (MFI%) reflects level of intracellular ROS. Higher MFI indicates more intracellular ROS. As shown in **Figure 1E**, DOX treatment could increase ROS in H9C2 cells, whereas DiOHF pretreatment attenuated the DOX-induced ROS production. Furthermore, we evaluated whether DiOHF can reverse the DOX-induced increase in ROS production. After DOX treatment for 2 h, we found that the increased ROS production was significantly reduced by the post-treatment with DiOHF (**Figure 1F**).

### DiOHF Prevented and Reversed DOX-Induced Cell Apoptosis

**Figure 2:**
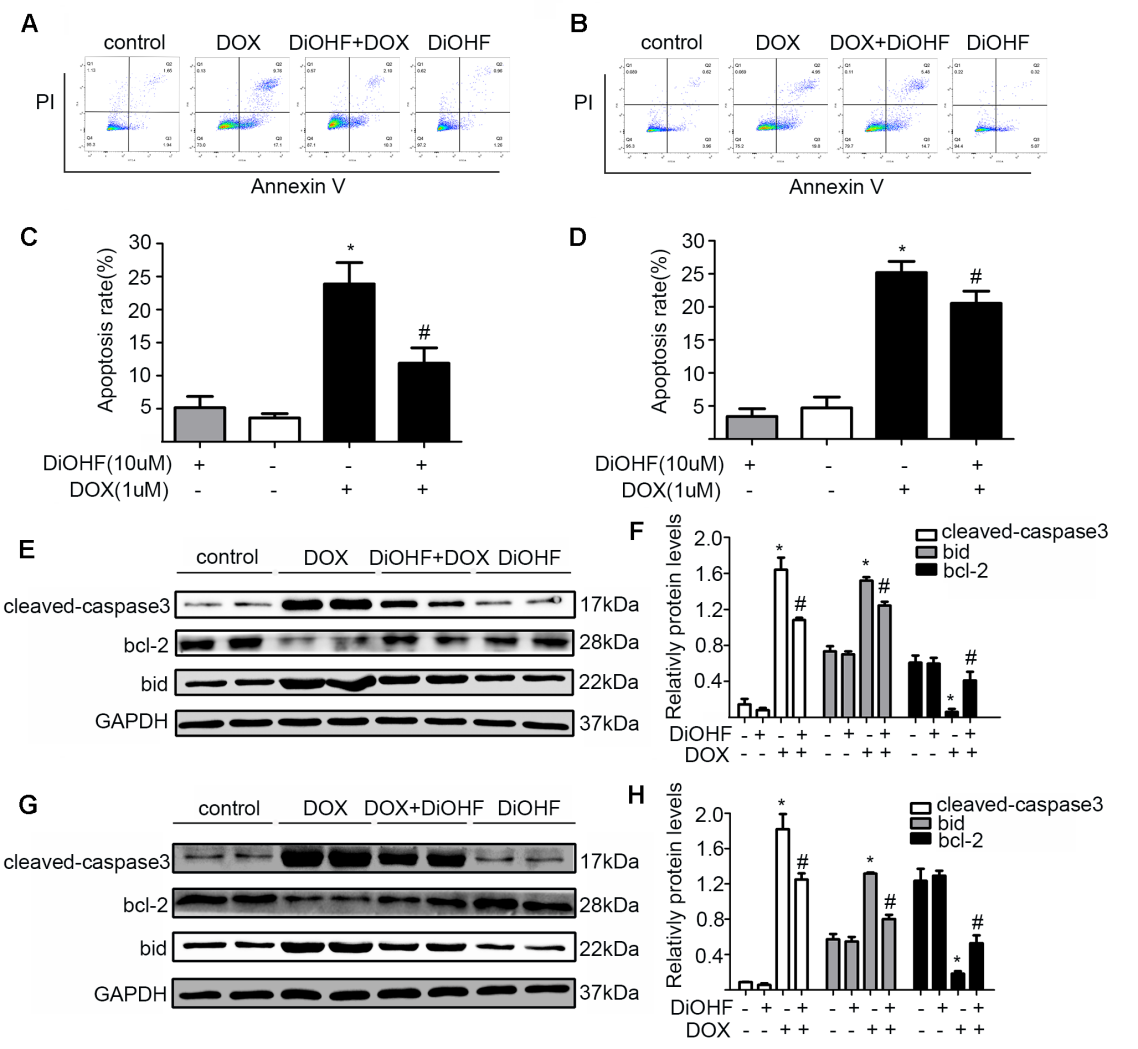
DiOHF prevented and reversed the DOX-induced apoptosis. **(A)** H9C2 cells were pre-treated with DiOHF for 2 h followed by the treatment with DOX for 24 h. Apoptosis was assessed by flow cytometry. **(B)** H9C2 cells were treated with DOX for 2 h followed by the treatment with DOX + DiOHF for 22 h. Apoptosis was detected by flow cytometry. **(C** and **D)** Quantitative data of the apoptosis. **(E** and **G)** Representative Western blots. **(F** and **H)** Western blot analysis for the apoptosis-associated proteins (cleaved-caspase-3, Bcl-2, and Bid). GAPDH served as the loading control. The data were analyzed by ANOVA and expressed as mean ± SEM. *P < 0.05, compared with the control; ^#^ *P* < 0.05, compared with DOX.

To assess the effects of DiOHF on DOX-induced apoptosis, H9C2 cells were sorted by a flow cytometry assay (FCM) after double labeling with Annexin V and propidium iodide. We found that the proportion of apoptotic cells significantly increased in the DOX treated cells (5.16 ± 1.70% in control vs 23.88 ± 3.23% in DOX group), and DiOHF pre-treatment (DiOHF + DOX group) inhibited the DOX-induced apoptosis (**Figures 2A, C**). Interestingly, the apoptosis could be largely reversed by 2 h post-treatment with DiOHF (**Figures 2B, D**). These findings indicate that DiOHF not only has a prophylactic effect, but also possibly plays a therapeutic role in the DOX-induced cell death. Cardiac apoptotic signaling pathways were also studied in terms of Bid, Bcl-2, and cleaved-caspase 3 expression. DOX treatment dramatically decreased the level of Bcl-2 and increased the level of cleaved-caspase 3 and Bid expression, which were reversed by pre- and post-treatment with DiOHF (**Figures 2E–H**).

### DiOHF Attenuated and Partially Recovered DOX-Induced Mitochondrial Dysfunction

**Figure 3:**
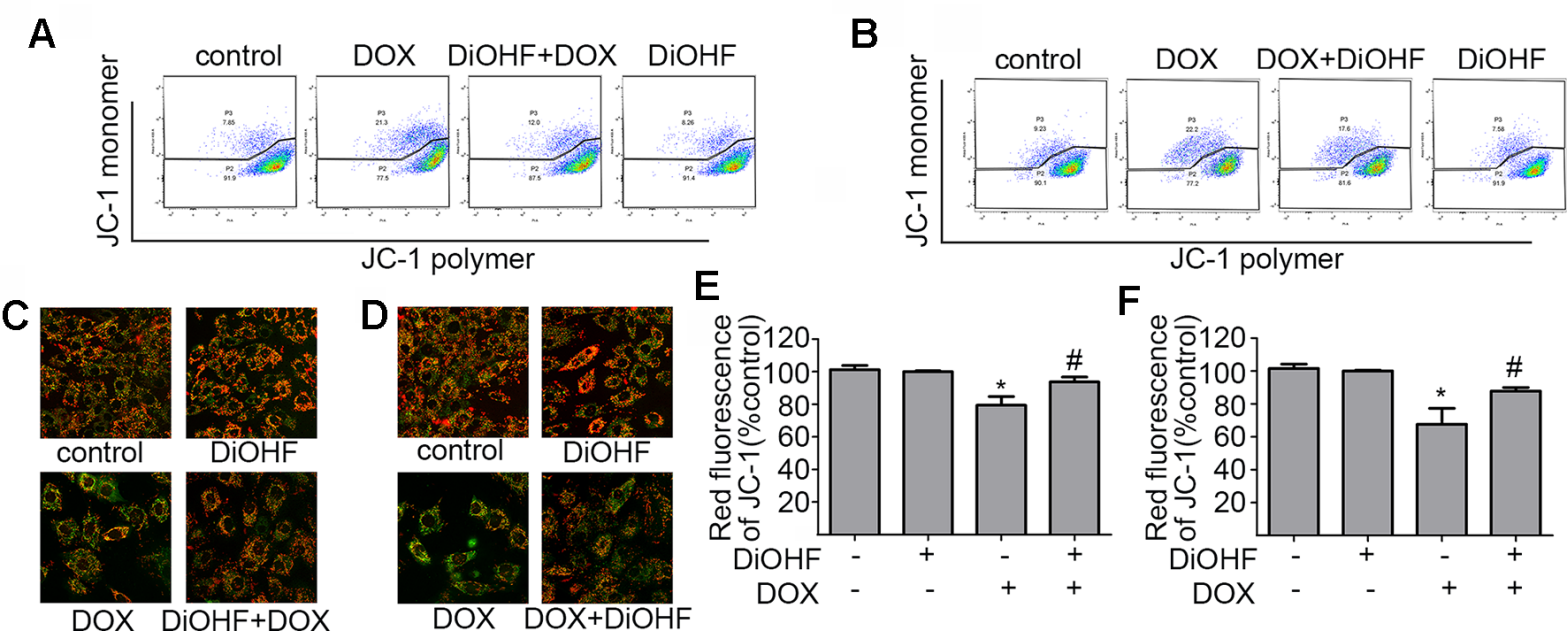
DiOHF prevented and rescued the DOX-induced mitochondrial dysfunction in H9C2 cells **(A** and **B)**. Effects of pre- and post-treatment of DiOHF (10 μM) on the loss of MMP in H9C2 cells treated with DOX (1 μM for 24 h; n = 3). JC-1 was observed as green monomers in the cytosol or as red aggregates in intact mitochondria. The change from red to green fluorescence indicated a collapse of mitochondria with intact membrane potential. The micrographs were recorded under confocal microscopy (Zeiss) at 40× magnification **(C** and **D**; n = 3**)**, and the red JC-1 fluorescence intensity was quantified **(E** and **F)** by Image Pro Plus. *P < 0.05, compared with control. ^#^P < 0.05, compared with DOX.

Maintenance of mitochondrial membrane potential (MMP) is critical for cell survival. MMP in H9C2 cardiomyocytes was determined by JC-1 staining and detected by FCM and laser confocal fluorescence microscopy (LCFM). JC-1 were observed as green monomers in the cytosol or as red aggregates in intact mitochondria. The change of fluorescence from red to green indicates loss of MMP. As shown in **Figure 3A**, DOX-exposure caused changes in JC-1 from polymer to monomer, which indicated a significant loss of MMP compared to the control. In addition, DiOHF pre-treatment could alleviate DOX-induced loss of MMP. In **Figure 3C**, the results acquired by confocal microscopy showed a decrease in red fluorescence and an increase in green fluorescence after DOX treatment, and a significant higher level of MMP was observed in cells that received DiOHF pre-treatment compared to that in cells treated with DOX alone. Then, we analyzed the red fluorescence ratio for the results of confocal microscopy by Image Pro Plus and found that the red fluorescence of JC-1 was significantly decreased compared to the controls (**Figure 3E**). However, DiOHF pre-treatment could alleviate DOX-induced changes, which was in line with the FCM results. We further investigated whether DiOHF could rescue the DOX-induced loss of MMP. The results (**Figures 3B, D, F**) showed that the red ratio was increased in the DiOHF post-treated cells compared to that in the DOX-treated cells, indicating that DiOHF could rescue the DOX-induced mitochondrial dysfunction.

### The Cardiac Protective Effect of DiOHF Was Mediated by ERK1 Activation

**Figure 4:**
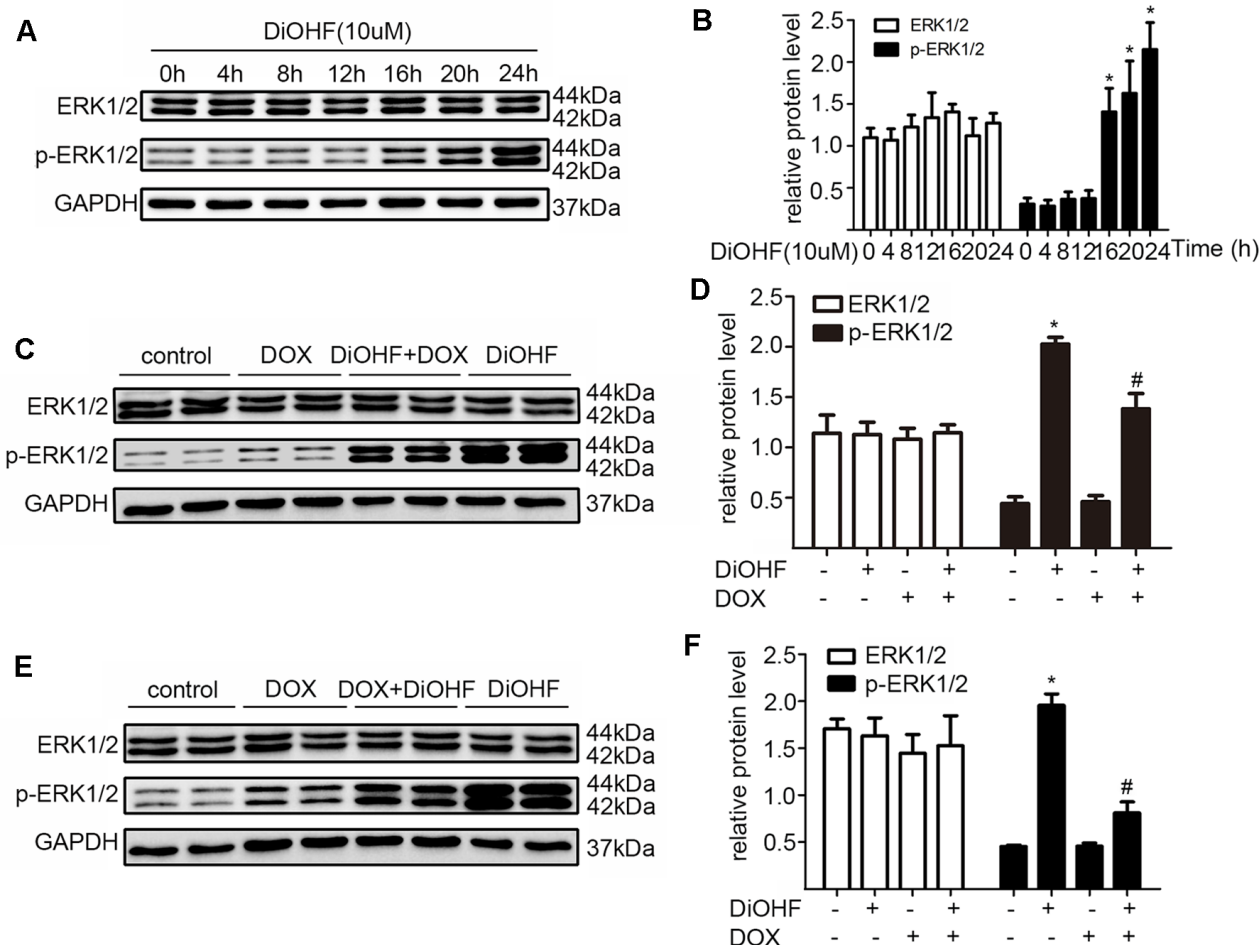
ERK1/2 participated in the protective effects of DiOHF. **(A)** DiOHF induced ERK1/2 activation in a time-dependent manner (n = 3). The H9C2 cells were treated with vehicle or DOX (1 µM) in the presence or absence of DiOHF (10 µM for 2 h prior to DOX exposure and 10 µM after 2 h of DOX exposure, respectively) for 24 h. The protein levels of phospho-ERK1/2 (p-ERK1/2) and ERK1/2 were measured by Western blot **(C** and **E**; n = 3**)**. **(B**, **D**, and **F**; n = 3**)** Quantitative results of ERK1/2 and p-ERK1/2. The data were analyzed by ANOVA and expressed as the mean ± SEM. *P < 0.05, compared with control. ^#^ *P* < 0.05, compared with DOX.

ERK1/2 signaling pathway was hypothesized to play an important role in protecting against myocardial injury. We first determined the effects of DiOHF on the activation of ERK1/2. Western blot analysis confirmed that DiOHF treatment dramatically increased p-ERK1/2 level in a time-dependent manner (**Figure 4A–B**) without changes in the total ERK1/2 expression, indicating an increased ERK1/2 phosphorylation. Besides, the level of p-ERK1/2 activated by DiOHF (pre- and post-treatment) was significantly higher than that activated by DOX (**Figures 4C–F**).

**Figure 5:**
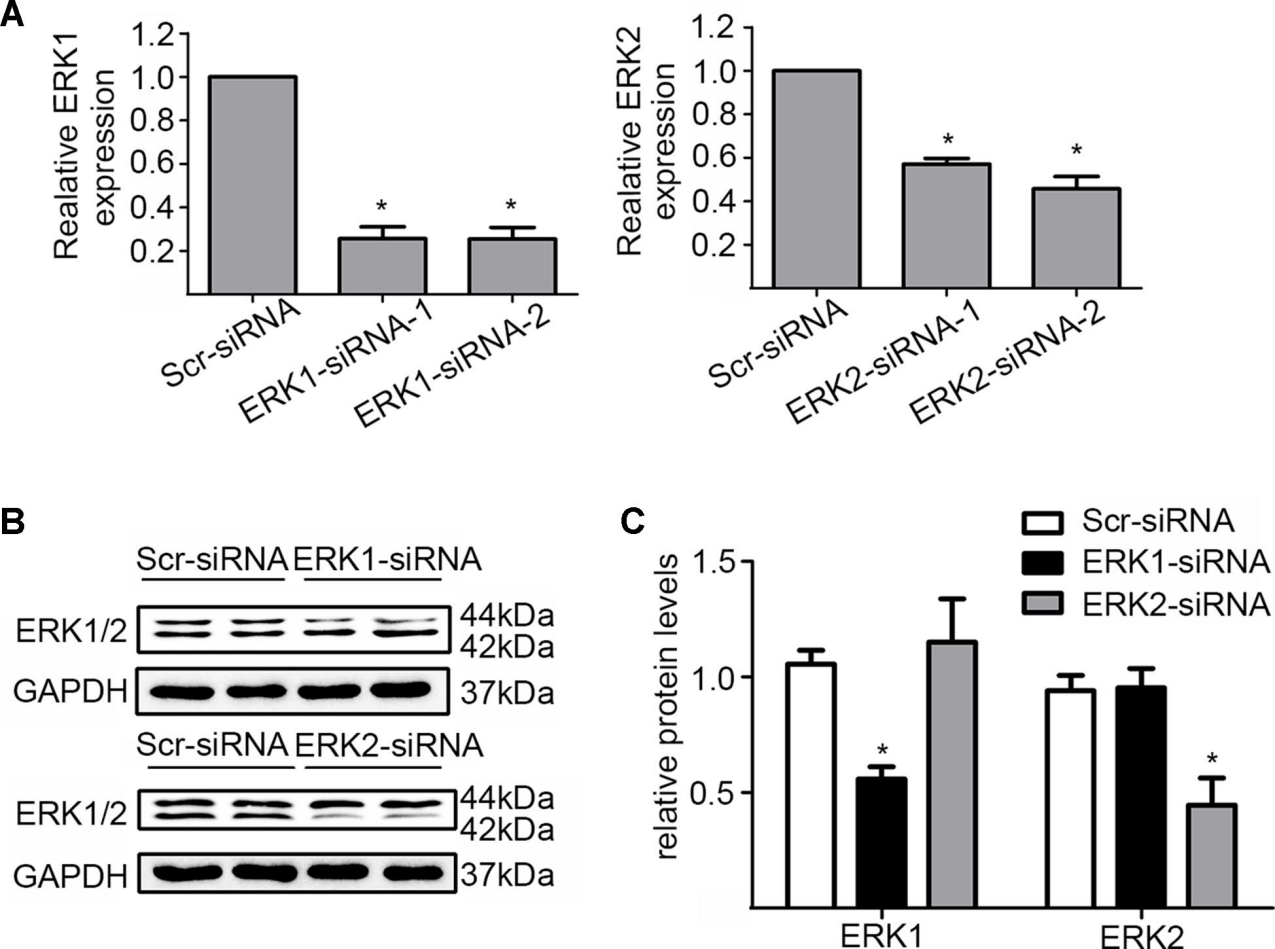
Knock-down of ERK1 or ERK2. **(A)** ERK1 or ERK2 silencing at the mRNA level (n = 3). **(B)** ERK1 or ERK2 silencing at the protein level (n = 3). **(C)** Quantitative results of ERK1 or ERK2 proteins (n = 3). The data were analyzed by ANOVA and expressed as the mean ± SEM. *P < 0.05, compared with Scr-siRNA.

**Figure 6:**
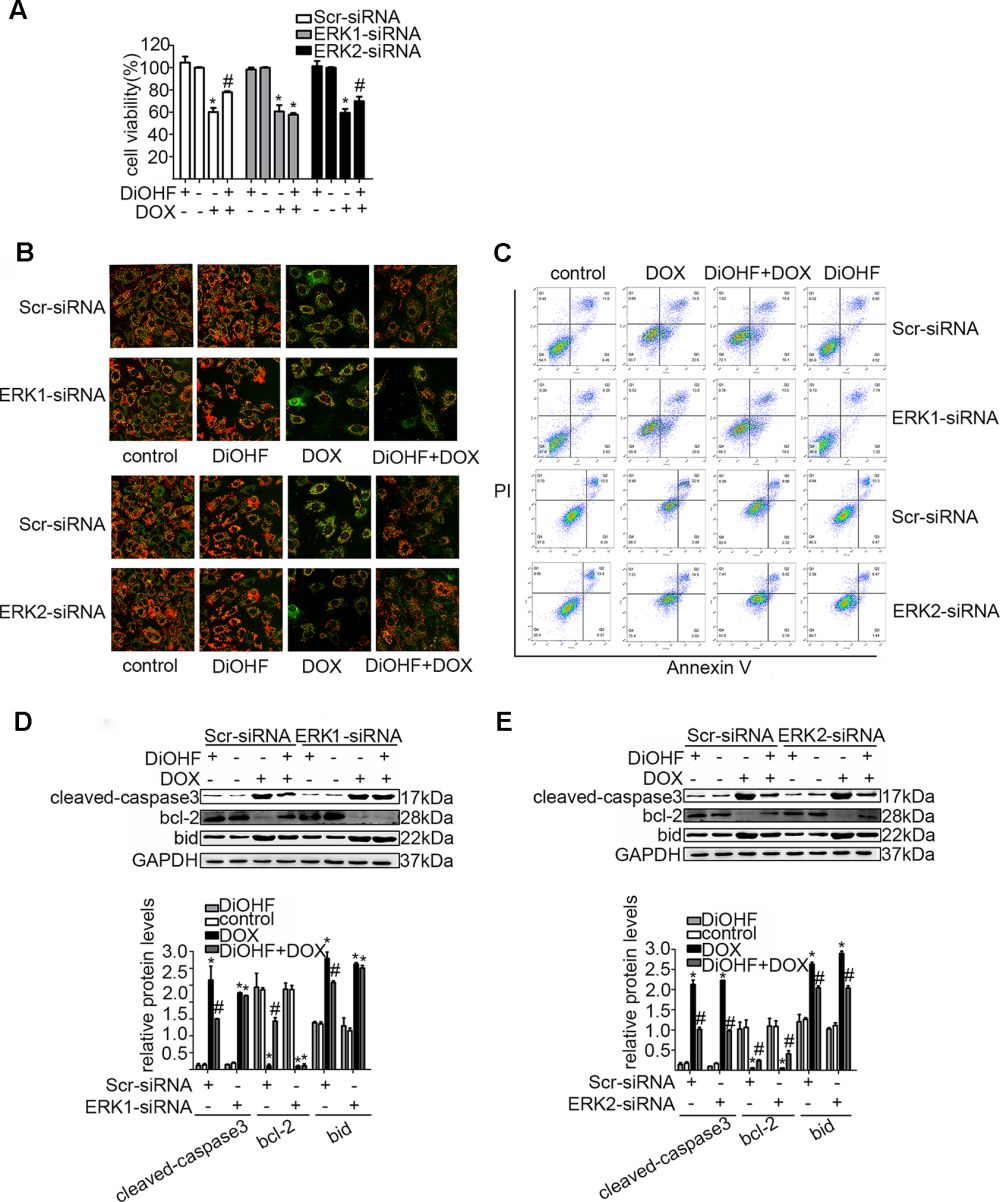
ERK1 siRNA abolished the cardioprotective effects of DiOHF against DOX-induced damage in H9C2 cells. Effects of ERK1 or ERK2 silencing on cell viability **(A**; n = 3), MMP **(B**; n = 3**)**, apoptosis rate **(C**; n = 3**)**, and apoptosis-associated proteins (cleaved-caspase-3, Bcl-2, and Bid) **(D** and **E)**. **(D** and **E)** Results of western blot and quantitative results of the apoptosis-associated proteins. The data were analyzed by ANOVA and expressed as the mean ± SEM. *P < 0.05, compared with control. ^#^ *P* < 0.05, compared with DOX.

In addition, we silenced the ERK1 and the ERK2 by ERK1-siRNA and ERK2-siRNA respectively to dissect their role in the DiOHF-associated cardioprotective effects. Both siRNAs showed specific and efficient knock-down of the distinct kinase (**Figure 5A–C**). ERK1 knock-down abolished the protective effects of DiOHF against DOX-induced reduction of cell viability (**Figure 6A**), mitochondrial dysfunction (**Figure 6B**), apoptosis (**Figure 6C**), and apoptosis-related protein (**Figures 6D, E**), while the ERK2 knock-down had no effect. These results demonstrated that the cardiac protective effects of DiOHF are mediated by ERK1 activation.

### DiOHF Suppressed the DOX-Induced Cardiotoxicity in Mice

**Figure 7:**
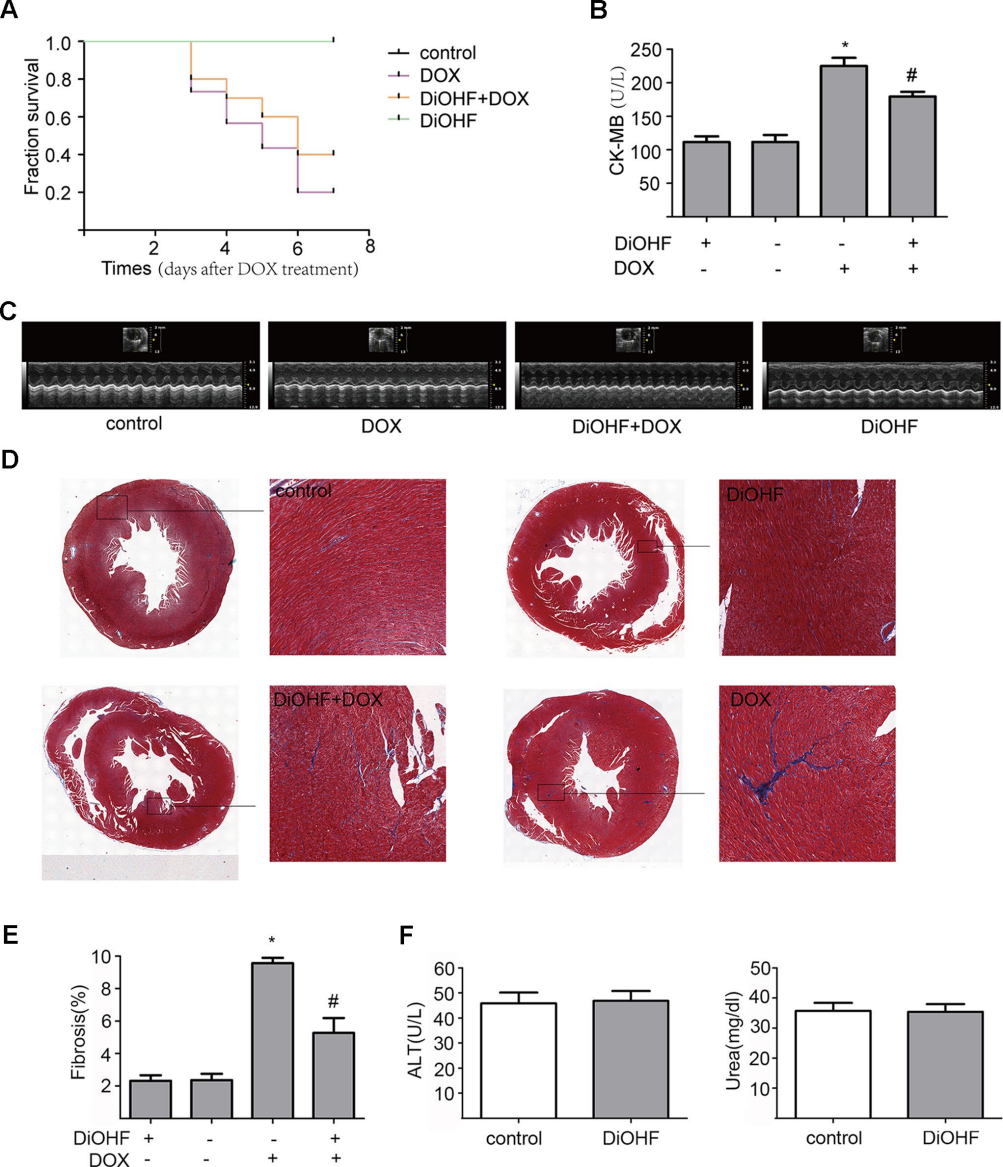
Protective effects of DiOHF against DOX-induced death, loss of body weight, cardiac dysfunction, and fibrosis in mice **(A)** Kaplan-Meier survival analysis in mice after vehicle or DOX administration with or without DiOHF pretreatment (p > 0.05; n = 30 for each group). **(B)** Serum CK-MB levels measured in all groups of mice. **(C)** Echocardiographic images acquired on the 7th day after DOX treatment in all the mice. **(D)** Representative Masson’s trichrome-stained photomicrographs exhibiting cross-sectional area of cardiomyocyte and myocardial fibrosis. **(E)** Quantitative analysis of the fibrotic areas (Masson’s trichrome-stained area in light blue normalized to the total myocardial area) obtained from 30–50 randomly chosen fields. **(F)** The levels of blood ALT (n = 7) and Urea (n = 9) in mice. *P<0.05, compared with control. ^#^P<0.05, compared with DOX.

We found that DiOHF could blunt or even reverse the DOX-induced cytotoxicity *in vitro*. To further explore the role of DiOHF in DOX-induced cardiotoxicity *in vivo*, Mice were observed daily after the administration of DOX (20 mg/kg i.p). The DOX-treated mice manifested weakness, lethargy, and lost weight, and a total of 87.10% of the mice died within 7 days of DOX administration (**Figure 7A**). DiOHF + DOX treatment, however, alleviated the above signs and reduced the mortality rate to 61.29%, and DiOHF alone caused no mortality. In addition, a significant decrease in body weight was observed in the DOX-treated mice compared to the controls, whereas DiOHF attenuated the DOX-induced weight loss (**Table 2**). Consistent with these findings, the serum CK-MB level was significantly increased (*P* < 0.05) on the 4^th^ day after DOX administration. But in the DiOHF + DOX group, CK-MB level was significantly lowered than that in the DOX group (**Figure 7B**) (*P* < 0.01).

**Table 2:** Body weight of mice.

Group	Body weight (1st day, g)	Body weight (7^th^ day, g)
Control	21.53 ± 0.81	24.34 ± 1.17
DOX	20.77 ± 1.02	15.91 ± 1.16*
DOX +DiOHF	20.79 ± 1.17	18.17 ± 1.62^^^
DiOHF	20.92 ± 0.90	24.31 ± 1.32

The data were analyzed by ANOVA and are expressed as the mean ± SEM. *P < 0.05 as compared to the control and ^^^P < 0.05 as compared to DOX.

Furthermore, cazrdiac function was monitored by echocardiography in all mice on the 7^th^ day after DOX treatment. As shown in **Figure 7C** and **Table 3**, mice treated with DiOHF alone did not show any changes in cardiac function. DOX administration impaired cardiac function characterized by the decrease in LVEF and LVFS and increase in LV vol; s and LVID; s. DiOHF pre-treatment largely blunted the DOX-induced cardiac dysfunction. In our study, we observed no significant changes in hypertrophy after DOX injection (**Supplementary Figure 3**).

**Table 3:** Ultrasound data statistics.

	Control (n=6)	DiOHF (n=6)	DOX (n=6)	DiOHF + DOX (n=6)
LVAW; d	0.80 ± 0.19	0.61 ± 0.25	0.76 ± 0.25	0.56 ± 0.08
LVAW; s	1.69 ± 0.17	1.52 ± 0.22	1.38 ± 0.37	1.45 ± 0.10
LVID; d	3.24 ± 0.25	3.32 ± 0.17	3.71 ± 0.22*	3.42 ± 0.09^#^
LVID; s	1.19 ± 0.18	1.19 ± 0.24	2.26 ± 0.20*	1.70 ± 0.17^#^
LVPW; d	0.58 ± 0.07	0.64 ± 0.08	0.45 ± 0.07*	0.61 ± 0.08^#^
LVPW; s	1.24 ± 0.08	1.36 ± 0.22	0.75 ± 0.20*	1.18 ± 0.16^#^
LV Mass (corrected)	53.95 ± 9.20	50.35 ± 12.59	58.74 ± 17.25	54.93 ± 12.51
LV Vol; d	42.51 ± 7.24	45.02 ± 5.82	58.84 ± 8.35*	47.98 ± 2.88^#^
LV Vol; s	3.40 ± 1.33	3.34 ± 1.81	17.46 ± 3.96*	8.69 ± 1.86^#^
FS%	63.20 ± 5.58	65.12 ± 5.86	39.27 ± 3.78*	50.90 ± 3.39^#^
EF%	91.88 ± 2.99	92.83 ± 3.19	70.43 ± 4.71*	82.92 ± 2.94^#^

*Represents p < 0.05 compared with control group; ^#^represents p < 0.05 compared with DOX group.

We also evaluated the fibrotic changes on the 7^th^ day after DOX treatment. As shown in **Figures 7D, E**, the fibrotic area in the DOX group was significantly increased (4-fold increase to the controls) and this change has been blunted by DiOHF pre-treatment. In line with the in vitro results, we found that DiOHF increased the protein level of ERK1/2 (**Supplementary Figure 2**) and reduced myocardial apoptosis (**Supplementary Figure 1**) after DOX treatment. Furthermore, to determine the possible *in vivo* toxicity of DiOHF, we examined the blood levels of ALT and urea in mice injected with DiOHF, we observed no changes in the ALT and urea (**Figure 7F**).

## Discussion

Anthracyclines, such as DOX, represent a pillar of many cancer treatment protocols, although they frequently induce cardiotoxic effects and their use is somewhat limited (Cappetta and De Angelis, 2017). To overcome this problem, two strategies are probably taken, either synthesizing a DOX analog with less toxicity or co-administration with cardioprotective agents. As the former strategy might reduce the DOX efficacy, combining DOX with a cardioprotective agent is likely more practical. Dexrazoxane is the only Food and Drug Administration (FDA) approved cardioprotective agent against DOX (Kane et al., 2008), but its use has been restricted because of its carcinogenic potential (Shaikh et al., 2016). Thus, it is important to look for new drugs based on the mechanism of DOX-induced cardiotoxicity.

DiOHF is a newly developed synthetic flavonoid. It has attracted much attention recently in terms of its cardioprotective properties in myocardial ischemia–reperfusion injury (Wang et al., 2004). Whether DiOHF can also protect against the DOX-induced cardiotoxicity has not been reported. Our present study demonstrated that DiOHF could not only protect against, but also reverse the DOX-induced cardiac injury *via* ERK1 activation. In line with the *in vitro* results, we confirmed *in vivo* that DiOHF effectively prevented the DOX-induced cardiotoxicity, including improvement of cardiac function and inhibition of myocardial fibrosis.

There is plenty of evidence showing that DOX-induced cardiotoxicity includes ROS production, mitochondrial dysfunction, and apoptosis (Dhalla et al., 2000; Green and Leeuwenburgh, 2002; Pereira et al., 2011). It has been established by several studies in different animal models that DiOHF has cardioprotective effects against myocardial ischemia–reperfusion injury (Wang et al., 2004; Qin et al., 2011; Woodman et al., 2014; Thomas et al., 2015; Chin et al., 2018). Similar to the cardiac ischemia–reperfusion injury, oxidative stress and cardiomyocyte apoptosis are also mediators for the DOX-induced cardiotoxicity (Dhalla et al., 2000). Our results demonstrated that DiOHF is a potent antioxidant against the DOX-induced oxidative stress *in vitro*, as evidenced by inhibition of ROS generation and improving cell viability. These results corroborated with the results reported by Wang et al. (Wang et al., 2004), who found that DiOHF markedly reduced NADPH-induced superoxide production, and its antioxidant properties relied on a catechol group in ring B and a 2,3-double bond conjugated with the 4-oxo group in ring C (Wang et al., 2004). Furthermore, Pietta also demonstrated that DiOHF could scavenge ROS by donating hydrogen atoms to free radicals (Pietta, 2000). The protective actions of DiOHF we observed are similar to those previously reported. In 2014, Woodman et al. found that DiOHF could inhibit mPTP opening and thus preserve mitochondrial function (Woodman et al., 2014). It is known that continuous opening of mPTP can result in apoptosis. In accordance with the previous studies, we found that DiOHF rescued the mitochondrial function in the DOX-induced cardiotoxicity. We elucidated that DiOHF stabilizes the MMP to protect mitochondrial function and prevent apoptosis. In line with the *in vitro* results, we observed amelioration of cardiac function and myocardial fibrosis *in vivo*.

ERK1/2, a mitogen-activated protein kinase (MAPK), has been reported to play an important role in the protection against myocardial injury (Hausenloy et al., 2005; Bhagatte et al., 2012; Hernandez-Resendiz and Zazueta, 2014). There is accumulating evidence that ERK is a pro-survival kinase which is closely related to mitochondrial mPTP opening and mitochondrial dysfunction (Hernandez-Resendiz and Zazueta, 2014). In addition, it has been reported that ERK1/2 activation is associated with the reduced intracellular ROS and apoptosis (Su et al., 2006; Xiang et al., 2009; Kim et al., 2014). Consistent with these findings, we observed activation of ERK1/2 in cells treated with DiOHF. However, some reports have identified differences between the ERK1 and ERK2 signaling (Shin et al., 2015; Verma et al., 2015; Luo et al., 2017). Verma et al. (Verma et al., 2015) demonstrated that decreased ERK1 phosphorylation could prevent Nrf2 from translocating into the nucleus, leading to generation of ROS and apoptosis. Luo et al. (Luo et al., 2017) reported roles of microRNA in regulating ERK1. They indicated that miR-15b-5p might target ERK1 to regulate proliferation and apoptosis (Luo et al., 2017). Both of the studies suggested that ERK1 was closely related to cell proliferation and apoptosis. On the other hand, Shin et al. (Shin et al., 2015) reported that activation of ERK2 promoted the low glucose-induced cell death (Shin et al., 2015). To dissect the individual role of ERK1 and ERK2 in cardiac protection against the DOX-induced cardiotoxicity, we silenced ERK1 or ERK2 respectively in H9C2 cells and confirmed that the protective mechanism of DiOHF was mediated by activation of ERK1 but not the ERK2.

To test whether DiOHF has a therapeutic effect on DOX-induced cardiotoxicity, we treated H9C2 cells with DiOHF after 2 h DOX incubation. The cell viability, mitochondrial function, and apoptosis were largely reversible by DiOHF treatment. However, the magnitude of recovery was, however, not as good as that of prevention by DiOHF pre-treatment. This might be due to the fact that DiOHF-induced activation of ERK1/2 is in a time-dependent manner (**Figure 4A**), and DOX-induced injury is a progressive process.

In summary, our study demonstrated that DiOHF effectively ameliorated the cardiotoxic effects of DOX both *in vitro* and *in vivo*. These could be attributed mainly to the inhibition of ROS production, preserving mitochondrial function and reducing apoptosis *via* activation of the ERK1. To our knowledge, this is the first study to reveal both the prophylactic and therapeutic effects of DiOHF on the DOX-induced cardiotoxicity. Although we did not observe any side effects of *in vivo* DiOHF application in terms of the functional liver or kidney injury, whether the insights gleaned from the pre-clinical models apply to human certainly needs further investigations.

## Data Availability

The raw data supporting the conclusions of this manuscript will be made available by the authors, without undue reservation, to any qualified researcher.

## Ethics Statement

This study was carried out in accordance with the Guide for the Care and Use of Laboratory Animals published by the US National Institutes of Health. The protocol was approved by the Animal Ethics Committee of Wuhan University.

## Author Contributions

DC contributed to the data acquisition, analysis, accuracy, integrity, and interpretation; drafting of the manuscript; HL and CQ were responsible for data analysis and integrity; YW was responsible for study concept and design; data interpretation, revision and approval of the manuscript. 

## Funding

This study was supported by grants from the National Natural Science Foundation of China to YW (NSFC, grants 81420108004, 81270304 and 81873507).

## Conflict of Interest Statement

The authors declare that the research was conducted in the absence of any commercial or financial relationships that could be construed as a potential conflict of interest.

## Supplementary Material

Figure S1 A was the Western Blotting results of heart tissues, and B was the histogram of the relative protein level of cleaved-caspase 3 (n = 3).

Figure S2 A was the Western Blotting results of heart tissues, and B was the histogram of the relative protein level (n = 3).

Figure S3 Cross-Sectional areas of cardiomyocytes for each group. We selected 50 individual cells per slide and the cell area sizes were calculated by pixel counting (n = 6 for each group).
