# Supplementary figures and images for: DiOHF Protects Against Doxorubicin-Induced Cardiotoxicity Through ERK1 Signaling Pathway
Source: Front Pharmacol. 2019 Sep 27;10:1081. doi: 10.3389/fphar.2019.01081 (PMC6777440; doi:10.3389/fphar.2019.01081)

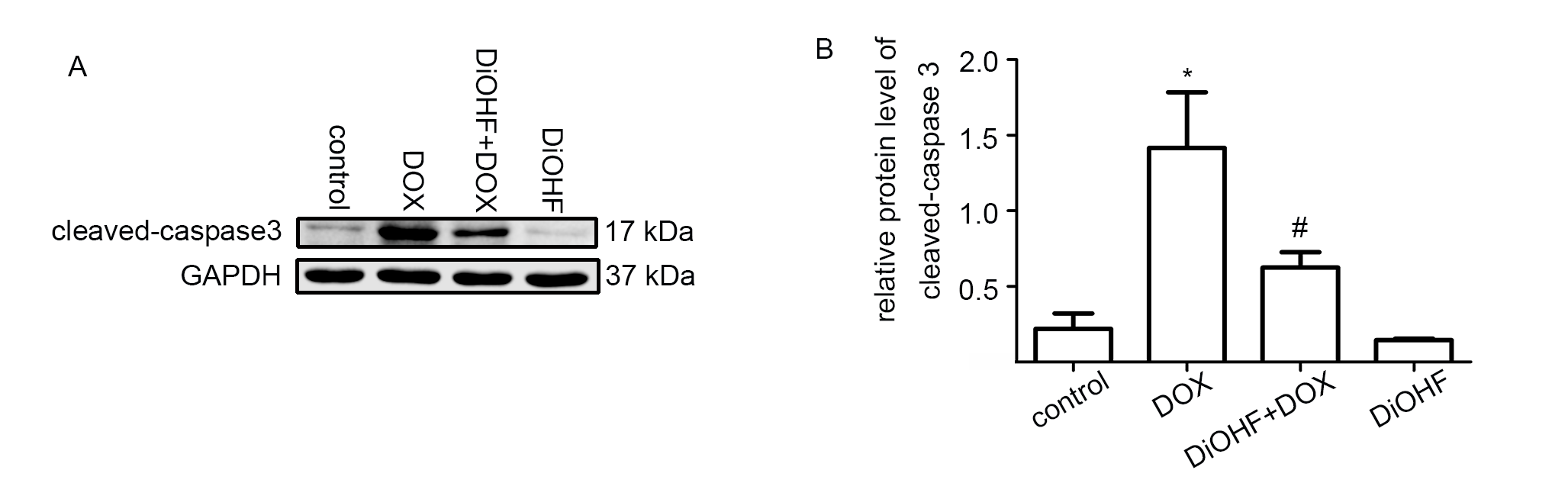

Supplement: Figure S1 — A was the Western Blotting results of heart tissues, and B was the histogram of the relative protein level of cleaved-caspase 3 (n = 3). [file Image_1.tif]

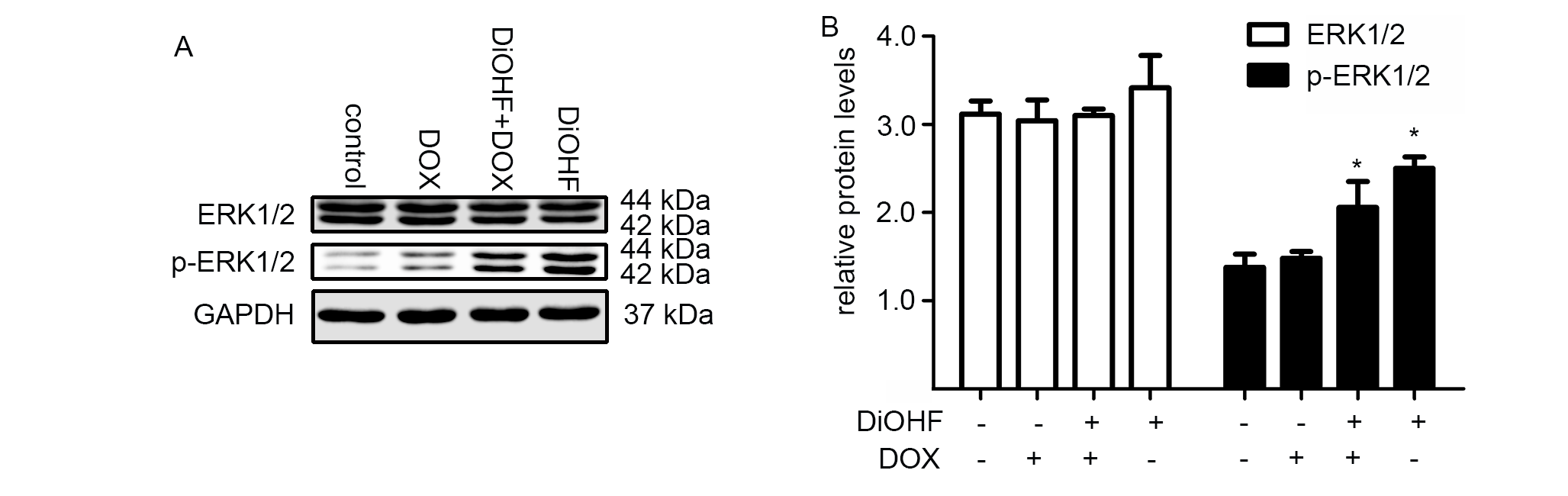

Supplement: Figure S2 — A was the Western Blotting results of heart tissues, and B was the histogram of the relative protein level (n = 3). [file Image_2.tif]

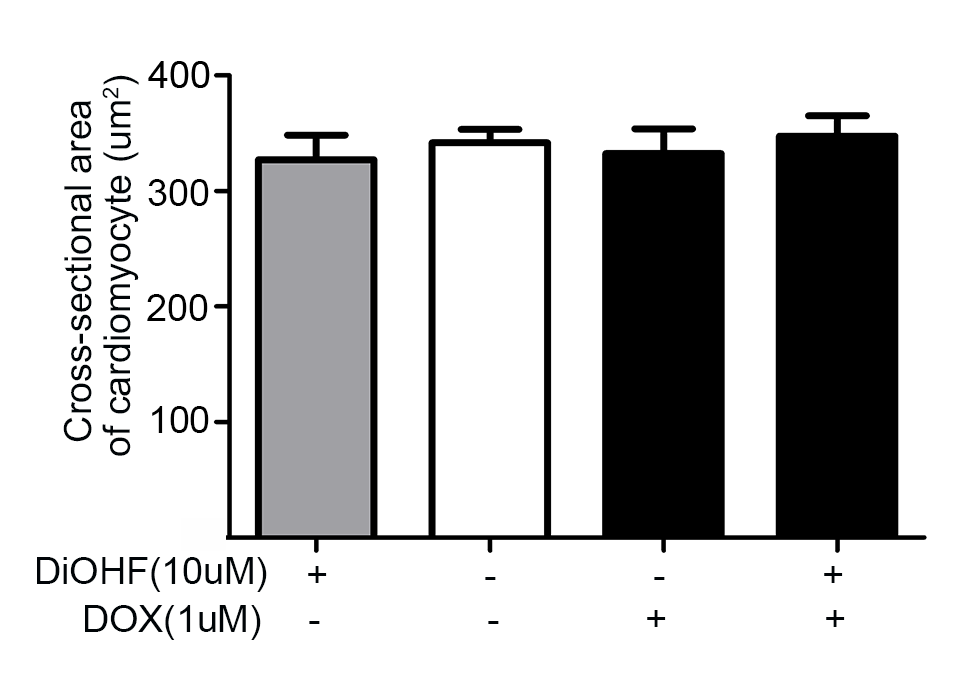

Supplement: Figure S3 — Cross-Sectional areas of cardiomyocytes for each group. We selected 50 individual cells per slide and the cell area sizes were calculated by pixel counting (n = 6 for each group). [file Image_3.tif]
